# Decreasing incidence of peptic ulcer complications after the introduction of the proton pump inhibitors, a study of the Swedish population from 1974–2002

**DOI:** 10.1186/1471-230X-9-25

**Published:** 2009-04-20

**Authors:** Michael Hermansson, Anders Ekedahl, Jonas Ranstam, Thomas Zilling

**Affiliations:** 1 Department of Surgery, Sahlgrenska University Hospital, Gothenburg, Sweden; 2 Department of Community Health Sciences, Lund University, Malmö, Sweden; 3 NKO, Lund University Hospital, Lund, Sweden; 4 Department of Surgery, Varberg Hospital, Sweden

## Abstract

**Background:**

Despite a decreasing incidence of peptic ulcer disease, most previous studies report a stabile incidence of ulcer complications. We wanted to investigate the incidence of peptic ulcer complications in Sweden before and after the introduction of the proton pump inhibitors (PPI) in 1988 and compare these data to the sales of non-steroid anti-inflammatory drugs (NSAID) and acetylsalicylic acid (ASA).

**Methods:**

All cases of gastric and duodenal ulcer complications diagnosed in Sweden from 1974 to 2002 were identified using the National hospital discharge register. Information on sales of ASA/NSAID was obtained from the National prescription survey.

**Results:**

When comparing the time-periods before and after 1988 we found a significantly lower incidence of peptic ulcer complications during the later period for both sexes (p < 0.001). Incidence rates varied from 1.5 to 7.8/100000 inhabitants/year regarding perforated peptic ulcers and from 5.2 to 40.2 regarding peptic ulcer bleeding. The number of sold daily dosages of prescribed NSAID/ASA tripled from 1975 to 2002. The number of prescribed sales to women was higher than to males. Sales of low-dose ASA also increased. The total volume of NSAID and ASA, i.e. over the counter sale and sold on prescription, increased by 28% during the same period.

**Conclusion:**

When comparing the periods before and after the introduction of the proton pump inhibitors we found a significant decrease in the incidence of peptic ulcer complications in the Swedish population after 1988 when PPI were introduced on the market. The cause of this decrease is most likely multifactorial, including smoking habits, NSAID consumption, prevalence of Helicobacter pylori and the introduction of PPI. Sales of prescribed NSAID/ASA increased, especially in middle-aged and elderly women. This fact seems to have had little effect on the incidence of peptic ulcer complications.

## Background

Peptic ulcer complications have a high mortality, especially in elderly patients [1] and it is therefore important to understand the epidemiology of this disease in order to investigate if complications can be prevented. Despite new efficient drugs to treat peptic ulcer disease and increasing knowledge about its aetiology, the incidence of peptic ulcer complications, i.e. perforation and bleeding, have been reported by several groups to be unchanged (table 1). However, in a previous study from Lund University Hospital we found a fall in the incidence of peptic ulcer perforation from 1974 to 1992 in our primary uptake area [1]. We wanted to investigate the incidence of peptic ulcer complications in a larger population before and after the introduction of the Proton pump inhibitors (PPI) in order to investigate whether the introduction of this ulcer healing drug has influenced the incidence of these diseases.

**Table 1:** Reported incidences of peptic ulcer complications (cases per 100000 inhabitants per year).

Country (ref)	Period	Reported incidences		Comment	Studied variable
		perforation	Bleeding		
**Norway (18)**	1935–1990	About 10		Report birth cohort specific risks for perforation	Admittance rates

**USA (44)**	1956–1985	10-5	7-4	Relatively stable incidence of emergency operations for PUC	Emergency operations

**England and** **Wales (13)**	1958–1962 1979–1982			Increasing incidence of PUC* and NSAID use in elderly ♀	National register

**Scotland (13)**	1958–1962 1979–1982			Increasing incidence of PUC* and NSAID use in elderly ♀	National register

**USA (45)**	1974–1976 1984-1984	No rate per 100000 inhab	No rate per 100000 inhab	13% increase in perforations and 7% increase in bleedings after H2-rec blocker introduction	Cases operated

**Poland (11)**	1977–1996	No rate per 100000 inhab		Constant number/year, increasing % elderly women, increasing mean age	Cases operated

**Hong-Kong (19)**	1979–1985	14–18			Operating room logbooks

**New South Wales (19)**	1979–1985	3–4			Diagnosis reported to dept of heath

**Finland (12,46)**	1972–1987 1987–1999	4–5 5–7	3–4	Increasingly more ♀, increasing mean age	Cases operated, national register

**Sweden (1**)	1974–92	2–11		Significant decrease in incidence	Patients records

**Denmark (20)**	1974–1984	4–10	5–10	No significant difference before/after H2-receptor blockers	Cases operated

**Finland (17)**	1977–1989	2–8	3–9	No significant difference before/after H2-receptor blockers	Cases operated

**Finland (15)**	1979-85-00	3-6-4		No significant difference before/after H2-receptor blockers or PPI	Patients records

**UK (10)**	1989–99	♂ 10–11 ♀ 7-7	27–31 14–16		Admittance rates

**UK (47)**	1996–98	♂ 5 ♀ 4		Only duodenal perforations, increasing mean age in ♀	Patients records

**Germany (23)**	1989–90 1999-00		51 49	Patients older and more NSAID use in the later period	Prospective

*PUC = peptic ulcer complications

Well-designed studies have clearly shown that NSAID and ASA contribute to the development of peptic ulcer disease and upper gastro-intestinal complications in a dose-dependent manner [2-10]

In a study from the United Kingdom, Walt et al reported increasing sales of NSAID during the 1970's and 1980's to elderly women, a cohort that has also been reported to have an increasing incidence of peptic ulcer complications [1,11-14]. Increased use of ASA and NSAID might influence the incidence of peptic ulcer complications over time. A recent Danish study report increased overall use of NSAID after the introduction of the selective COX-2 inhibitors in 1999 [15]. This phenomenon coincided with a stable hospitalization rate of peptic ulcer bleeding but a decrease in hospitalization for perforated peptic ulcer in their study. We wanted to study if a correlation between sales of NSAID and complications to peptic ulcer complications could be found in the Swedish population and thus we further studied the sales of prescribed ASA and NSAID in Sweden during the same period.

## Methods

The population of Sweden increased from 8,2 millions in 1974 to 8,9 millions in 2002. The Swedish national health system is tax funded giving the whole population unrestricted access to health services.

### Data on incidence of ulcer complications

In this study all cases of gastric and duodenal ulcer complications diagnosed in Sweden from 1974 to 2002 were identified using the National Hospital Discharge Register (National Board of Health and Welfare, S-10630, Stockholm, Sweden). Since 1987 it is mandatory for hospitals to report to this register. In practise, however, it has been in existence since the early seventies though not in the entire country. We compensated for this when the incidence figures from 1974 to 1986 were calculated, as described below.

The ICD codes used for the search are listed in table 2. In ICD 8 and ICD 9 there are codes named ".99" or ".9" in the last positions which represent "unspecified location without knowledge of bleeding or perforation". These codes does not exist in ICD 10 which means that the patients that were earlier coded as totally unspecified are now included in other codes. This fact could mean that incidences of ulcer complications are underestimated before 1997 when ICD 10 was introduced. During the period 1974–86 between 30–37% of all reported codes for peptic ulcer disease were reported as "totally unspecified". From 1987 to1996 this number was 16–20% according to the National Department of Health. Codes representing ulcer with bleeding as well as perforation are included in both the bleeding and the perforation groups.

**Table 2:** Codes searched for in the national register

Ulcer complication	ICD 8 (-1986)	ICD 9 (1987–96)	ICD 10 (1997-)
Perforated gastric ulcer	53100, 53101	5311, 5312 5315, 5316	K251, K252 K255, K256

Perforated duodenal ulcer	53200	5321, 5322 5325, 5326	K261, K262 K265, K266

Other perforated ulcer	53300, 53400	5331, 5332 5335, 5336 5341, 5342 5345, 5346	K271, K272 K275, K276 K281, K282 K285, K286

Bleeding gastric ulcer	53190, 53193	5310, 5312 5314, 5316	K250, K252 K254, K256

Bleeding duodenal ulcer	53290	5320, 5322 5324, 5326	K260, K262 K264, K266

Other bleeding ulcer	53390, 53490	5330, 5332 5334, 5336 5340, 5342 5344, 5346	K270, K272 K274, K276 K280, K282 K284, K286

The data from the National Department of Health are reliable. In a study from 1994 regarding validity, they found that in 11–14% of the cases the diagnosis reported was not correct if they applied a very strict judgement, i.e. all five figures correct [16]. If they were slightly less stringent, i.e. three figures correct, the inaccurate diagnoses was about 6–8%.

### Data on sales of NSAID/ASA

Information on sales of NSAID was obtained from the National Prescription Survey (The National Corporation of Swedish Pharmacies – Apoteket AB). Data on drugs dispensed on prescription during the period 1975 – 1986 used a sample of 1/288 of all dispensed prescriptions. From 1987 – 2002, the sample was 1/25 (4%) of all dispensed prescriptions. However, medication to patients in Nursing Homes, Hospices etc. is only included if their medication had been dispensed on prescription by the pharmacies.

The composition of the combination-product Treo Comp^® ^was changed in 1985, and the ATC-code was changed accordingly from N02BA71 (ASA, combinations with psycholeptics) to N02AA59 (codeine combinations). The DDD for Treo Comp^® ^is 3 tablets, corresponding to 1,5 g ASA, but the DDD for ASA is 3 g. One DDD of Treo Comp^® ^thus corresponds to 0,5 DDD ASA. The figures for Treo Comp^® ^have been recalculated to DDD's of ASA.

### Statistics

Age-standardised incidence rates were calculated using the Swedish population in 1995 as an external reference. Yearly site, age and gender specific incidence rates were calculated using data from the national population register. Time trends were investigated using linear regression analysis with log(incidence) rates as the dependent variable and the calendar year as an independent variable. An exploratory spline regression analysis of log incidence with 6 uniformly distributed knots was also performed using a piecewise linear function for time. STATA 10.1 was used for the statistical calculations. All p-values are two-sided and p-values below 5% were considered statistically significant.

The ethical committee of Lund University Hospital approved the study.

## Results

### Age Standardised incidence

#### Perforated Peptic Ulcer in Males

**Figure 1:**
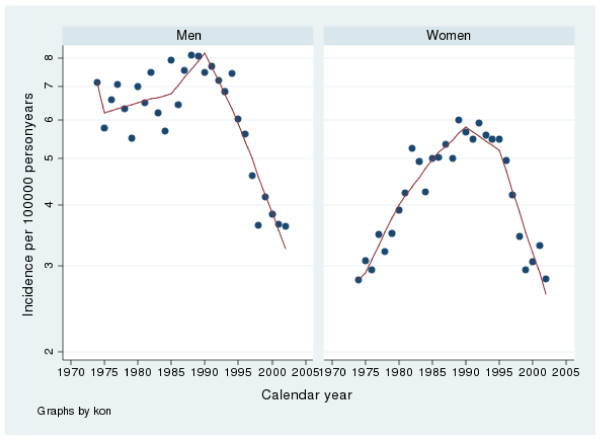
**Incidence of gastric perforated peptic ulcers in Sweden from 1974 to 2002**. Note that the y-axis has been truncated.

**Figure 2:**
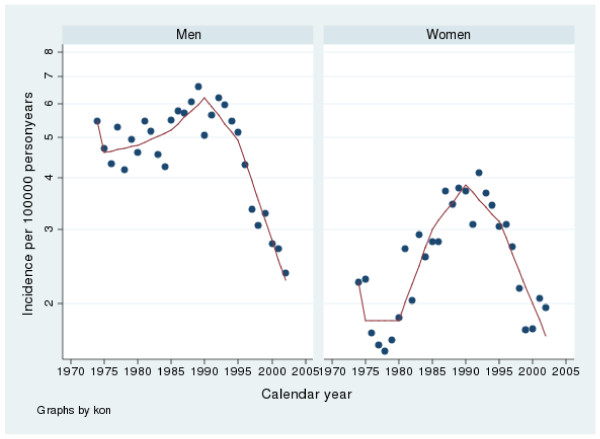
**Incidence of duodenal perforated peptic ulcers in Sweden from 1974 to 2002**. Note that the y-axis has been truncated.

The incidence of gastric perforation varied from 3.5 to 7.8 and for duodenal perforation from 2.3 to 6.4 per100 000 inhabitants per year. There were slightly more gastric perforations throughout the whole period. From 1974 to 1988 there was a rise in incidence (p < 0.002) and from 1988 to 2002, a fall in incidence (p < 0.001) according to the linear regression analysis. The spline regression analyses regarding perforations in males are presented in tables 3 and 4 as well as in figures 1 and 2. The results are similar to the linear regression analysis.

**Table 3:** Spline regression analysis, Gastric perforated ulcers

	Males		Females	
Year	**Coef**.	95% Conf. interval	**Coef**.	95% Conf. interval
**1975**	-0.14	-0.41 0.13	0.03	-0.19 0.25

**1980**	0.01	-0.04 0.05	0.06	0.03 0.10

**1985**	0.01	-0.03 0.05	0.04	0.01 0.08

**1990**	0.04	-0.001 0.08	0.03	-0.003 0.06

**1995**	-0.07	-0.1 -0.03	-0.02	-0.05 0.01

**2000**	-0.08	-0.11 -0.05	-0.10	-0.12 -0.07

**Table 4:** Spline regression analysis, Duodenal perforated ulcers

	Males		Females	
Year	**Coef**.	95% Conf. interval	**Coef**.	95% Conf. interval
**1975**	-0.17	-0.44 0.09	-0.21	-0.58 0.16

**1980**	0.01	-0.04 0.05	-0.00	-0.06 0.06

**1985**	0.02	-0.02 0.06	0.10	0.04 0.16

**1990**	0.03	-0.004 0.07	0.05	-0.01 0.10

**1995**	-0.05	-0.09 -0.01	-0.04	-0.09 0.01

**2000**	-0.11	-0.14 -0.08	-0.09	0.13 0.05

#### Bleeding Peptic Ulcer in Males

**Figure 3:**
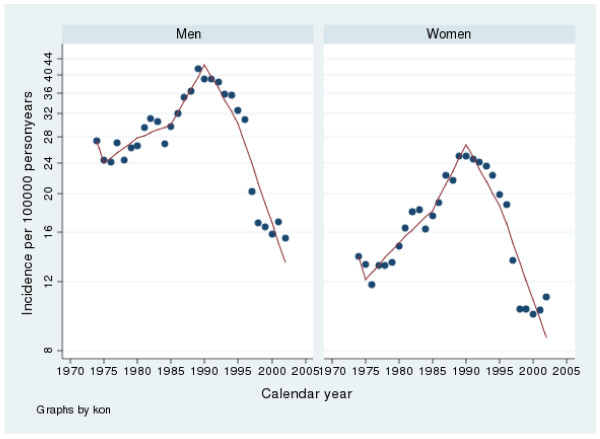
**Incidence of gastric bleeding peptic ulcers in Sweden from 1974 to 2002**. Note that the y-axis has been truncated.

**Figure 4:**
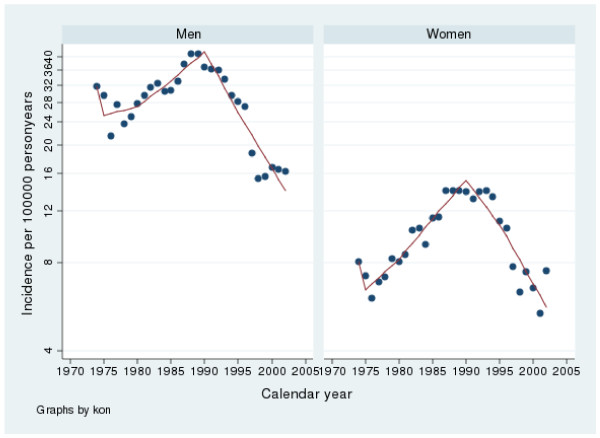
**Incidence of duodenal bleeding peptic ulcers in Sweden from 1974 to 2002**. Note that the y-axis has been truncated.

The incidence of bleeding gastric ulcer varied from 14.9 to 40.2 and for bleeding duodenal ulcer between 15.0 to 40.0 per100 000 inhabitants per year. From 1974 to 1988 there was a rise in incidence (p < 0.001) and from 1988 and 2002 a fall in incidence (p < 0.001) according to the linear regression analysis. The spline regression analyses regarding bleeding in males are presented in tables 5 and 6 as well as in figures 3 and 4. The results are similar to the linear regression analysis

**Table 5:** Spline regression analysis, Gastric bleeding ulcers

	Males		Females	
Year	**Coef**.	95% Conf. interval	**Coef**.	95% Conf. interval
**1975**	-0.13	-0.38 0.12	-0.14	-0.42 0.15

**1980**	0.03	-0.14 0.07	0.04	-0.005 0.09

**1985**	0.01	-0.02 0.53	0.04	-0.005 0.08

**1990**	0.07	0.03 0.11	0.08	0.03 0.12

**1995**	-0.07	-0.10 -0.03	-0.07	-0.11 -0.03

**2000**	-0.12	-0.14 -0.09	-0.11	-0.14 -0.08

**Table 6:** Spline regression analysis, Duodenal bleeding ulcers

	Males		Females	
Year	**Coef**.	95% Conf. interval	**Coef**.	95% Conf. interval
**1975**	-0.23	-0.52 0.06	-0.23	-0.53 0.08

**1980**	0.01	-0.03 0.06	0.05	-0.004 0.10

**1985**	0.04	-0.004 0.08	0.06	0.02 0.11

**1990**	0.05	0.003 0.09	0.06	0.01 0.11

**1995**	-0.10	-0.14 -0.05	-0.07	-0.11 -0.02

**2000**	-0.09	-0.12 -0.05	-0.09	-0.13 -0.06

#### Perforated Peptic Ulcer in Females

The incidence of gastric perforation varied between 2.7 to 5.8 and for duodenal perforation from 1.5 to 4.0 per 100 000 inhabitants per year. There were relatively more gastric than duodenal perforations during the whole period. Between 1974 and 1988 there was an increase in incidence (p < 0.001) and from 1988–2002 a fall in incidence (p < 0.001) according to the linear regression analysis. The spline regression analyses regarding perforations in females are presented in tables 3 and 4 as well as in figures 1 and 2. The results are similar to the linear regression analysis

#### Bleeding Peptic Ulcers in Females

The incidence of bleeding gastric ulcers varied between 9.6 and 24.2 and for bleeding duodenal ulcers from 5.2 to13.6 per100 000 inhabitants per year in females. There were relatively more gastric than duodenal bleeding ulcers during the whole period. Between 1974 and 1988 there was an increase in incidence (p < 0.001) and from 1988 to 2002 a decrease in incidence (p < 0.001) according to the linear regression analysis. The spline regression analyses regarding bleeding in males are presented in tables 5 and 6 as well as in figures 3 and 4. The results are similar to the linear regression analysis

### Sales of NSAID and ASA

**Figure 5:**
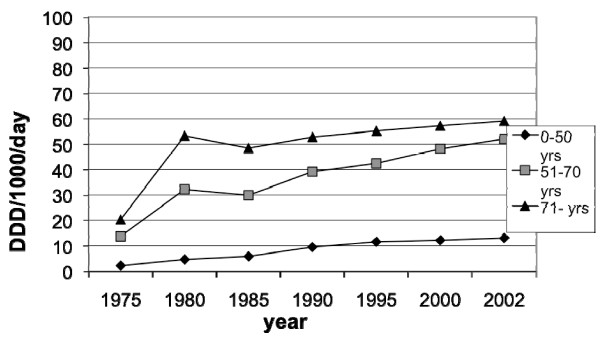
**Sales of prescribed NSAID and ASA between 1975–2002 in Sweden among males**.

**Figure 6:**
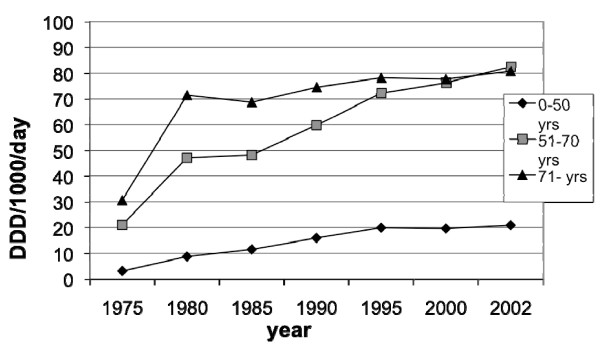
**Sales of prescribed NSAID and ASA between 1975–2002 in Sweden among females**.

**Figure 7:**
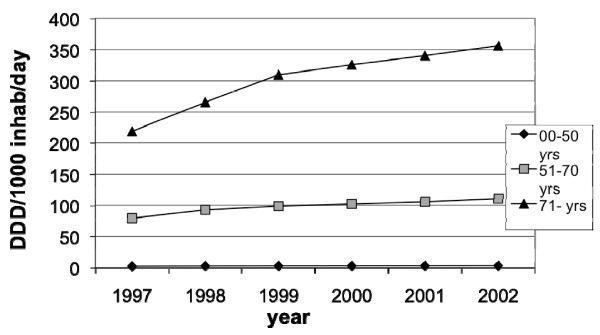
**Sales of prescribed low-dose ASA between 1997–2002 in Sweden among males**.

**Figure 8:**
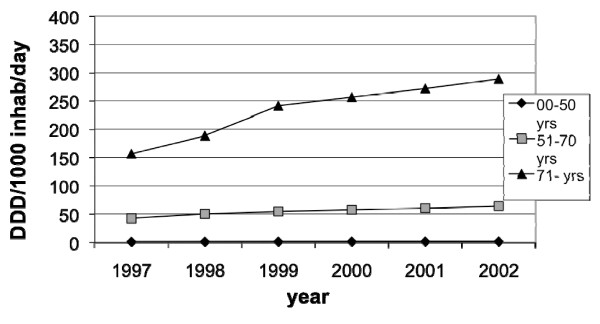
**Sales of prescribed low-dose ASA between 1997–2002 in Sweden among females**.

**Figure 9:**
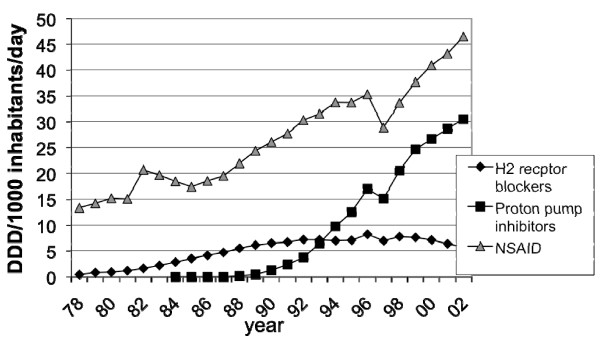
**Sales of gastric acid inhibitors and NSAID:s in Sweden 1978–2002**.

The sales of prescribed NSAID and ASA in Sweden for the years 1975 through 2002 are presented in figures 5, 6, 7, 8 and 9. In males the sales of NSAID and ASA described as DDD per1000 inhabitants per day rose from 2.3 to13.1 in the age group 0–50 years, from 13.7 to 52.1 in the age group 51–70 years and from 20.3–59.1 in the age group 71–99 years.

The corresponding figures for females were from 3.2 to 20.9 in the age group 0–50 years, from 21.0 to 82.5 in the age group 51–70 years and from 30.6 to 80.8 in the age group 71–99 years.

The sales to females compared to males were higher in all age groups and the difference increased towards the end of the study period.

The total sales of prescribed NSAID/ASA (DDD per1000 inhabitants per day) increased from 13.3 in 1978 to 46.5 in 2002.

The total volume of NSAID and ASA (DDD per1000 inhabitants per day) sold both "over the counter" and prescription sales, increased from 45.0 in 1978 to 57.6 in 2002 according to the statistics of Apoteksbolaget AB, Sweden. This means that sales increased 28% during this period.

The sales of low-dose ASA (DDD per1000 inhabitants per day) in males increased from 2.75 to 3.55 in the age group 0–50 years, from 79.2 to 110.9 in the age group 51–70 years and from 219.0–356.2 in the age group 71–99 years (fig 7).

The sales of low-dose ASA (DDD per1000 inhabitants per day) in females increased from 1.5 to 2.0 in the age group 0–50 years, from 42.3 to 64.2 in the age group 51–70 years and from 156.4–288.9 in the age group 71–99 years (fig 8).

### Sales of acid inhibitors

H2 receptor blockers were introduced in Sweden in 1978. In 2002 total sales were 5.7 DDD per1000 inhabitants per day (figure 9).

The first PPI were introduced in Sweden in 1988. In 2002 total sales were 30.5 DDD per1000 inhabitants per day (figure 9).

### Mean age

In the whole material mean age rose from 59.0 years to 67.9 years from 1974 to 2002. The mean age in males with gastric complications rose from 59 years in 1974 to 66.5 years in 2002 The corresponding figures for females were 63.2 and 70.2 years.

The mean age in males with duodenal complications rose from 52.8 years in 1974 to 62.1 years in 2002. In females the corresponding figures were 61.1 and 72.6.

## Discussion

In the mid-eighties two events took place that changed the treatment of peptic ulcer patients. First the discovery of the connection between Helicobacter pylori and peptic ulcer disease and second the introduction of the first PPI in 1988. However, despite new and efficient treatment regimes several authors report that the incidence of complications of peptic ulcer disease, perforation and bleeding, has been relatively unchanged during the last thirty years of the 20-th century [1,11-14,17-26]. In contrast we found a significant fall in incidence of both bleeding and perforated ulcers in the Swedish population after 1988 despite increasing sales of ASA and NSAID, drugs known to be ulcerogenic.

### Incidence of peptic ulcer complications

According to the literature, the incidence of peptic ulcer complications appears to be relatively stable over the last three decades with approximately 5 to10 episodes per 100 000 inhabitants/year. Some relevant references are listed in table 1. In elderly women, several authors even report an increasing incidence [11-14,27]. At least three studies have compared the incidences of peptic ulcer perforation before and after the introduction of the H2-receptor antagonists but found the incidence to be unchanged [1,22,23]. In contrast to these studies, we report a significant decrease in peptic ulcer complications in the Swedish population over the last two decades. Recently, a Danish group report similar results from 1996 to 2004 regarding perforated peptic ulcer but not for bleeding ulcers [15]. Like us they had a relatively large sample size, the Danish study involved a population of 1, 2 million inhabitants. A plausible explanation for the fact that several studies fail to detect any differences might be that the studied populations have often been too small for a disease that has a relatively low incidence to begin with. Differences in the prevalence of the different risk factors for peptic ulcer complications between populations, which will be discussed below, are other possible explanations.

The significant drop in incidence of peptic ulcer complications reported in this study, can possibly be explained by a generational (cohort) influence, for example less exposure to Helicobacter pylori infections among younger generations compared with older birth cohorts. Another possibility is a secular (periodical) phenomenon, for example the influence of extensive exposure of acid-suppressing drugs or changing trends in the use of factors known to cause ulcer or counteract ulcer healing, such as tobacco, alcohol or ulcerogenic drugs. Further, the use of gastroscopy has probably increased during the study period in Sweden enabling the detection and treatment of symptomatic peptic ulcers before complications occur. In our opinion, the explanation for this uniform decline in complications after 1988 is most likely multifactorial.

In the sections below we will discuss some important risk factors for complicated peptic ulcer disease and their possible influence on our results. When interpreting the results one must be aware that this is an ecological study with risk of "ecological fallacy" [28]. The connections discussed below can, of course, be caused by other factors and should therefore be adopted with caution.

### NSAID as a risk factor and influence of PPI

We found an increase in the sales of prescribed NSAID during the study period. The number of prescriptions increased with age and was highest among middle-aged and elderly women. As mentioned earlier, elderly women are also the group in which the incidences of peptic ulcer complications are reported to be increasing relative to other groups [1,11-14]. However, in conjunction with an increase of prescribed sales there was a fall in non-prescribed sales, resulting in a total increase in NSAID sales of 28% during this period.

Several studies support the notion that NSAID is a risk factor not only in uncomplicated peptic ulcer disease, but also in regard to perforated and bleeding ulcers [2,3,5-7,10,29,30]. A systematic review of 18 case-control studies from 1990 to 1999 reported a pooled RR of 3.6 [2]. The higher risk was maintained during treatment and disappeared after treatment termination. The added risk is dose-dependent and also includes low-dose ASA. Studies report higher risk with all doses, with a RR = 2 for bleeding ulcer with daily intake of 75 mg ASA [8,9]. According to a study by Sorensen et al from 2000 the risk increase further when low-dose ASA is combined with NSAID [10].

Bearing this in mind one would expect a rise in incidence of peptic ulcer complications when the sales of NSAID increases, which is quit contrary to our findings. Since ours is a descriptive study one can only speculate as to the relationship between peptic ulcer complications and NSAID use. In a recent German paper from 2005 Ohmann et. al. found no difference in the incidence of peptic ulcer bleeding when comparing the years 1989 and 1999 despite significantly higher NSAID consumption in the latter year [26]. Hawkey conducted a prospective, controlled study of patients with bleeding ulcers and found that *H pylori*-positive NSAID users had twice the risk compared to *H pylori *negative patients [31]. The prevalence of *H pylori *is declining and thus could have reduced the effect of increased NSAID use [32-34].

In the end of the 1990-ies the less ulcerogenic selective cyclo-oxygenase-2 (COX-2) inhibitors were introduced in Sweden. These drugs were not included in this study since they were only available for use in Sweden during the last years of the study period. Christensen et al report a 44% rise in prescriptions of all NSAID after 1997, mainly due to COX-2 inhibitors [15]. The hospitalization rates of peptic ulcer complications after the introduction of COX-2 inhibitors were, however, stable for bleeding ulcers and decreased from 17 to 12/100000 person-years from 1996 to 2004 for perforations. This indicates that the COX-2 selective drugs might have decreased the risk of peptic ulcer complications for the individual NSAID user.

Proton pump inhibitors (PPI) have become one of the most sold drugs in the world and are nowadays also available over the counter in Sweden. It is a well known fact that PPI protect against peptic ulcer complications in NSAID users [3,35-38]. Interestingly the introduction of PPI was almost simultaneous with the beginning of a falling incidence in peptic ulcer complications (fig 1, 2, 3, 4). We believe that the extensive use of PPI in the population of Sweden has reduced the incidence of peptic ulcer complications. This conclusion is supported by Vonkeman et al (2007) who performed a nested case-control study from a cohort of 52000 NSAID users [39]. They found a significant risk reduction regarding peptic ulcer complications in NSAID users who used PPI concomitantly. Selective COX-2 inhibitors did not reduce the risk in their study.

### Is Helicobacter pylori a risk factor in complicated peptic ulcer disease?

In the present study we found a significant decrease in peptic ulcer complications in the mid-eighties that co-inside with the discovery of H *pylori *as a cause for peptic ulcer disease. *H pylori *is an important pathogenic factor in uncomplicated peptic ulcer disease, although studies that investigate the connection between *H pylori *and peptic ulcer complications are somewhat divergent. For example, Gisbert et. al. (2003) calculated a mean prevalence of 68% from 19 studies with a range from 0–100% [40].

We have found five studies comparing patients with peptic ulcer complication patients to patients with non-GI related diseases in the control group. In three of these reports there was no difference in the prevalence of *H pylori *[5,41-44]. Three studies have compared patients with peptic ulcer complications patients to patients with uncomplicated ulcer disease [43,45,46]. Surprisingly, the prevalence of *H pylori *was slightly higher in the latter group.

*Helicobacter Pylori *is, without a doubt, connected to peptic ulcer disease. In the pathogenesis of perforation and bleeding, however, other factors such as NSAID and smoking are of great importance as well. In the time period covered by this study, the prevalence of *H pylori *infections has decreased in the western world [47] as has the incidence of peptic ulcer complications in our study (fig 1, 2, 3, 4). Since this is a descriptive study one can only speculate as to whether these two phenomena are correlated or not. There is however no doubt in the literature that eradication of *H pylori *is mandatory after a peptic ulcer complication, if the patient is *H pylori *positive. Otherwise the risk of recurrent ulcer disease is high [48-50].

### What about smoking?

Smoking is a risk factor for peptic ulcer perforation [5,18,51]. A prospective cohort study from Denmark found that smoking > 15 cigarettes daily increased the risk of peptic ulcer perforation 3,5 times [51]. A retrospective study from Norway report that a cohort pattern in prevalence of smoking partly explained the cohort pattern in perforation risk for both sexes [18]. The prevalence of smoking has declined during the last twenty years in Sweden, especially in men, which could perhaps account for fewer ulcer complications [52].

## Conclusion

When comparing the periods before and after the introduction of the proton pump inhibitors we found a significant decrease in the incidence of peptic ulcer complications in the Swedish population after 1988 when PPI were introduced on the market. The cause of the decrease is most likely multifactorial, including smoking habits, NSAID consumption, prevalence of Helicobacter pylori and the introduction of the PPI.

Patients with peptic ulcer complications are in average ten years older in 2002 than twenty years earlier. Sales of prescribed NSAID/ASA increased, especially in middle-aged and elderly women. This fact seems to have had little effect on the incidence of peptic ulcer complications, possibly because of a protective role of the PPI.

## Competing interests

This study was supported financially by Astra-Zeneca AB.

## Authors' contributions

Michael Hermansson was involved in the design of the study and in the collection of data. He has also written the paper. Anders Ekedahl has collected the information from the National Prescription Survey and helped with the interpretation of the results. Jonas Ranstam has performed the statistical calculations and helped with the interpretation of the results. Thomas Zilling has designed the study and helped with the editing of the paper and the interpretation of the results. All authors have read and approved of the manuscript.

## Pre-publication history

The pre-publication history for this paper can be accessed here:

http://www.biomedcentral.com/1471-230X/9/25/prepub
